# ‘Grey nomad’ travellers’ use of remote health services in Australia: a qualitative enquiry of hospital managers’ perspectives

**DOI:** 10.1186/s12913-022-07580-8

**Published:** 2022-02-05

**Authors:** Margaret Yates, Lin Perry, Jenny Onyx, Tracy Levett-Jones

**Affiliations:** 1 School of Nursing and Midwifery, Faculty of Health, University of Technology Sydney, Broadway, Ultimo, NSW 2000 Australia; 2 South East Sydney Local Health District and School of Nursing and Midwifery, Faculty of Health, University of Technology Sydney, Broadway, Ultimo, NSW 2000 Australia; 3 Business School, University of Technology Sydney, Broadway, Ultimo, NSW 2000 Australia; 4 School of Nursing and Midwifery, Faculty of Health, University of Technology Sydney, Broadway, Ultimo, NSW 2000 Australia

**Keywords:** Nursing managers, Health services, Hospitals, Travellers, Rural and remote, Grey nomads, Healthcare needs, Australia, Qualitative study

## Abstract

**Background:**

For more than the last two decades, older Australians travelling domestically in self-sufficient accommodation and recreational vehicles for extended periods of time have been referred to as ‘Grey Nomads’. By 2021 more than 750,000 such recreational vehicles were registered in Australia. Tourism data for the year to September 2017 show 11.8 million domestic camping and caravanning trips in Australia, 29% of which were people aged 55 and over. As the ‘baby boomer’ generation increasingly comes to retirement, the size of this travelling population is growing. This term applies to the spike in birth rates after World War II from 1946–1964. This growing group of domestic travellers are potential healthcare consumers in remote areas but relatively little is known about their travel, healthcare needs or care seeking practices. Grey nomads have been described as reflective of the age-comparable sector of the Australian population in that many live with chronic illness. Early concerns were raised that they may “burden” already stretched rural and remote healthcare services but relatively little is known about the impact of these travellers.

**Methods:**

The aim of this study was to explore the utilisation of healthcare services in remote locations in Australia by grey nomads including women travellers, from the perspective of healthcare professionals working in these settings. The study objective was to interview healthcare professionals to seek their experience and details of service delivery to grey nomads.

In March 2020 [prior to state border closures due to the COVID-19 pandemic] a field study was conducted to identify the impact of grey nomads on healthcare services in remote New South Wales and Queensland. A qualitative approach was taken to explore the perspectives of nursing healthcare managers working in remote towns along a popular travel route. With appropriate Research Ethics Committee approval, managers were purposively sampled and sample size was determined by data saturation. Thirteen managers were contacted and twelve interviews were scheduled to take place face to face in the healthcare facilities (small hospitals with acute care and aged care services) at mutually convenient times. A semi-structured interview schedule was developed in line with the research aim. The interviews were audio-recorded, transcribed and thematic analysis was undertaken concurrently with data collection for ongoing refinement of questions and to address emerging issues.

**Results:**

These nursing managers described a strong service and community ethos. They regarded travellers’ healthcare needs no differently to those of local people and described their strong commitment to the provision of healthcare services for their local communities, applying an inclusive definition of community. Traveller presentations were described as predominantly exacerbations of chronic illness such as chest pain, medication-related attendances, and accidents and injuries. No hospital activity data for traveller presentations were available as no reports were routinely generated. Travellers were reported as not always having realistic expectations about what healthcare is available in remote areas and arriving with mixed levels of preparedness. Most travellers were said to be well-prepared for their travel and self-management of their health. However, the healthcare services that can be provided in rural and remote areas needed to be better understood by travellers from metropolitan areas and their urban healthcare providers.

**Conclusion:**

Participants did not perceive travellers as a burden on health services but recommendations were made regarding their expectations and preparedness. Australia’s national transition to electronic health records including a patient—held record was identified as a future support for continuity of care for travellers and to facilitate treatment planning. With no current information to characterise traveller presentations, routinely collected hospital data could be extracted to characterise this patient population, their presentations and the resources required to meet their care needs.

**Supplementary Information:**

The online version contains supplementary material available at 10.1186/s12913-022-07580-8.

## Introduction

For at least the last two decades, older Australians travelling domestically for extended periods of time have been referred to as ‘Grey Nomads’. This term is applied to retired or semi-retired individuals, usually 50 years of age or older, who tour within Australia in caravans, campervans, motorhomes and the like for a minimum of three months. Onyx and Leonard [1] referred to the emergence of grey nomads in Australia as a phenomenon: a remarkable occurrence of people travelling for extended periods in self-sufficient accommodation vehicles. Perhaps due to the increasing retirement of the large post World War II ‘Baby Boomer’ generation, and more recently the travel restrictions imposed for the COVID-19 pandemic, traveller numbers are growing. Between 2011–2016, caravan and campervan registration numbers grew by 30% and 20%, respectively, totalling 615,301 for the year to January 2016 [2]; by 2021 more than 750,000 such recreational vehicles were registered [3]. Tourism data for the year to September 2017 showed 11.8 million domestic camping and caravanning trips in Australia, 29% of which were people aged 55 and over [4]. These travellers are potential healthcare consumers in rural and remote areas but relatively little is known about their travel patterns, healthcare needs and healthcare seeking practices [5].

Previous work has characterised grey nomads as reflective of the age-comparable sector of the Australian population in that many live with one or more chronic illnesses [6]. Tate et al. [7], for example, reported 55% of travellers had chronic disease and 70% took regular medication. Halcomb et al. [8] surveyed 316 long-term travellers and reported 40% with hypertension, 23% with arthritis, 30% with diabetes and other health conditions; almost one-quarter required ongoing prescription medication and almost half reported that their illness affected their daily life. Interviewing eight grey nomads with at least one chronic condition, Calma et al. [9] found them aware of their health needs and what was required for self-management, and that they ‘planned ahead’ to manage their health. Similarly, in interviews with diabetes educators in South Australia and fourteen travellers with a cancer diagnosis, both Abigail, McCloud [10] and Stephens, Halcomb [11] reported them as generally well organised: arranging their travel to dovetail with their health needs and planning ahead to ensure they took with them medications they required, or to ensure their availability.

However, concerns about the impact of these travellers on rural and remote health services persist [7, 9, 12]. Originating from what the author described as “largely anecdotal” reportage [13], this has not been tested or supported by primary data. This has implications for the provision of health services, policy, health infrastructure, education, and workforce planning in rural and remote communities.

### Study aim and objective

The aim of this study was to explore the utilisation of healthcare services in remote locations in Australia by grey nomads, including women travellers, from the perspective of healthcare professionals working in these settings. The study objective was to interview healthcare professionals to seek their experience of service delivery to grey nomads, to identify what services are sought, how this is managed, and what impact, if any, this has on local health services.

## Methods

### Study design

As one component of a larger study of women travellers, in March 2020 a field study was conducted to identify the impact of grey nomads, including women travellers, on healthcare services in remote New South Wales and Queensland. A qualitative descriptive approach was employed [14], chosen to explore the experiences and perspectives of healthcare professionals working in remote towns along a popular travel route frequented by grey nomads. Semi-structured interviews were chosen to explore each individual’s understanding [15] of this aspect of the familiar, day to day reality of care delivery at their facilities.

### Fieldwork settings

The study sites were all in small towns on the highway situated in areas nationally designated as remote and very remote in NSW and Queensland [16]. At the time of the field study, in March 2020, a five-year drought had recently broken with significant flooding which had cut roads and isolated towns. The towns visited included a mix of tourist destinations and locations without any particular attractions but rather fuel stops or suitable for an overnight stay. All are classified as small towns by size [17], with populations ranging from 335 to 3,335 people. Population decline in their towns was described by participants with the exception of two towns which were expanding.

The facilities managed by participants were all in the above small towns. Facilities were a combination of acute hospital and aged care beds, and community services. All had less than twenty acute hospital inpatient beds; the majority less than ten. Most of the beds were designated for aged care, with small Emergency Departments (ED) mainly comprised of two beds. The NSW facilities also had chairs for renal dialysis (Table 1). At the time of the interviews, the participants were being briefed on State COVID-19 planning and what was required for their facilities.

**Table 1:** Bed capacity for study site facilities

Facility Number	Total Inpatient Beds	Acute/Sub-Acute Care	Emergency Department	High Dependency	Short Stay	Aged Care	Renal Dialysis Chairs
1	42	6				36	4
2	35	11	2	2		15	2
3	20	8				12	6
4	16	8				8	8
5	30	4	4		2	20	
6	24		2				
8	8	4	2	2	1		
9	8		2			6	
10	19	15	4				
11	17	8	3			6	
12	13	1	2		2	8	

### Participants

The sample comprised healthcare managers responsible for the running of these facilities. Sampling employed a combination of purposive and convenience strategies. Two researchers [xx and xx] planned visits to the work locations of participants, who were identified as knowledgeable on this topic. A minimum of one healthcare manager per location was purposively sampled, recruiting those who were available for interview at a mutually convenient time. Sample size was determined by data saturation; that is, sampling continued until no new information was observed [18, 19]. The managers were contacted using publicly available information, provided with study details and invited to interview.

### Data collection and analysis

Face to face interviews were conducted wherever possible, and by telephone where it was not. The interviews took place at the healthcare facilities at mutually convenient times. Interviews were digitally audio recorded with consent, using an Olympus DM-720 Business recorder and a mobile phone application for backup; audio recordings were maintained on a secure cloud server. The interview schedule was developed in line with the research aim, discussed and refined amongst the author group. With local health services as the context, participants were asked about local services provided for the community and for grey nomads including women travelers; why grey nomads present to health care services, what they expect and how the health service responds; attitudes towards grey nomads accessing the health services and their presence in the community.

The interviews were transcribed verbatim and imported into NVivo Version 12 for thematic analysis. Analysis was undertaken concurrently with data collection to enable ongoing refinement of questions and follow up of emerging issues. The Braun and Clarke [20] six-step framework was used which included: becoming familiar with the data during the verbatim transcription of interviews and repeated readings of the transcripts; the generation of initial codes i.e., particular ideas or points of interest; collating relevant data under codes; the collating of codes under possible overarching themes and the identification of relationships amongst the themes and the ongoing analysis of the data within the themes. Analysis was undertaken by two authors [xx and xx] and findings were discussed and agreed to consensus amongst the author group.

To support the rigor of this study and demonstrate dependability and confirmability an audit trail was maintained of project activities. Frequent discussions with supervisors supported reflexivity [21].

### Ethics approval

The authors confirm that study methods were carried out in accordance with relevant guidelines and regulations including the COREQ guidelines. With the approval of the appropriate Human Research Ethics Committee (registration number ETH19-4565), written or verbal informed consent, where possible, or otherwise audio recorded verbal consent by telephone, was obtained. Confidentiality was maintained with use of code numbers in the transcribed files.

## Results

### Participants

Thirteen facility managers were contacted and twelve responded to the invitation for interview; eleven were conducted face to face and were approximately one hour long; one telephone interview took approximately twenty minutes. All the managers interviewed were nurses, women and responsible for the management of at least one hospital, and in some instances also community services. Participants explained how their facilities functioned to provide emergency care and recounted their experiences of traveller presentations. No participant had ready access to any reports of hospital routine data on traveller presentations; all accounts were based on their experiences and recollections.

Thematic analysis identified three key themes: services in and for the community, met and unmet expectations, and preparations.

### Services in and for the community

This broad theme encompassed an explanation of the healthcare and support services that were provided at the sites (and some of the challenges faced) and the ethos and culture of care for which these participants were responsible.

In describing their culture of care, all participants clearly expressed that they did not differentiate between local people and travellers in relation to how they are managed. Participants stressed the function of their facilities as serving their communities:

“We are a community hospital, and we are here to serve the community” [HCP8] and

“… I could get sick, you’re sick, that’s OK that’s what we are here for..”. [HCP12].

They also acknowledged the contributions of travellers to their local economies, for instance when they stop to re-fuel their vehicles, stay overnight or longer, purchase groceries and visit local coffee and gift shops, museums and art galleries.

Participants explained that services were provided on a holistic basis, which could be interpreted more broadly than is generally the case with metropolitan health services. Managers recounted events where the hospital staff had provided care that was extended to meet the needs of their patients’ partners and vehicles and even their dog (see Supplementary File 1, Case Example One). This was not viewed as anything out of the ordinary but part and parcel of serving their community, which was what they considered the basis of their role. The care provided was no different for grey nomads than for local community members.

“*We treat them as we treat our community or anybody... we don’t treat them any different to anybody else” [HCP12].*

Nonetheless, all the managers described the recruitment of nursing (and other) staff as a particular challenge in maintaining service delivery and all were heavily reliant on overseas nurses. They expressed disappointment that, unlike for teachers and police officers [in NSW], few incentives were available for nurses to work in remote and very remote areas.

*“We always have a recruitment problem… there’s a perception that everyone needs to be really experienced to work and that’s not necessarily the case. The remoteness of and the aging population of our nurses has become a very intricate weave” [HCP3].*

Participants went on to describe the services delivered at their facilities. They explained that they provide emergency care but are not equipped for longer term inpatient care. In the one nurse-led facility, they were able to keep patients overnight or for a couple of days as the highly skilled nurses could implement nursing interventions, within their scope of practice, to address conditions such as dehydration or mildly unstable diabetes. Telehealth was described at this site, so a doctor could talk directly to the patients. At all sites, emergency care was provided via high resolution digital cameras. Generally, an emergency services doctor was on the other end of the camera reviewing the patient and giving instructions for treatment. In other instances, retrieval services and their networks of specialties were accessed. For example,

*“We had a penetrating eye injury a few years ago where an ophthalmology surgeon told us what to do to avoid optical nerve damage… If someone’s had a stroke and they are in ED … instead of getting an ED doctor, we get a cardiovascular consultant to talk us through” [HCP11].*

Patients were generally airlifted to larger facilities when their illnesses could not be adequately treated locally. Where tertiary care was required, the patient could be stabilised onsite and then retrieved by the Royal Flying Doctor Services (RFDS).

Some participants expressed their personal purpose as the pursuit of service rather than career development. For some, but not all, this was linked to having grown up in the area.

“I try to put back into the community and to help others that need it” [HCP2].

*“You just wouldn’t come for climbing the corporate ladder type of thing and I mean, I grew up here, so it’s because you want to give the best back to the people who live in your community, and I mean I live here I grew up here so I’ve got that connection” [HCP3].*

### Traveller presentations

Participants discussed the frequency and characteristics of grey nomad presentations to their facilities.

The initial point of contact for accessing health services in these remote towns was the hospital emergency departments. With the exception of two participants, the managers agreed that few grey nomads presented to their EDs, and presentations were not often. One manager reported they could increase the ED throughput by about 10% during the main travelling season. Another stated that grey nomads doubled the number of presentations to their ED. This manager had been in the area only six months and requested a telephone interview due to her workload related to COVID-19; she did not elaborate in response to questioning, during what was the briefest of the interviews. The other participants reported the number of presentations fluctuated and are seasonal, depending on what activities were happening locally. They described significant events attracting travellers during the annual seasonal migration north between March—October (although most events were cancelled in 2020 and 2021 due to the COVID-19 pandemic).

Participants reported that generally more men than women travellers were seen in their EDs. They commented that while they might see solo women in the towns, they seldom presented to the hospital. One manager described a convoy of women passing through the town on their way to an event but reported that travellers were predominantly heterosexual couples. However, no site data were available to support these accounts, which derived from their recollections.

When asked about the impact of traveller presentations, participants said that, as numbers were small, the overall impact on their services was no different to that of local people. One participant recounted that grey nomads presented for the same reasons as local people: mainly accidents and exacerbation of chronic illness. This reinforced the view of grey nomads as no different to the local community in terms of service need and demand. No-one reported hospitals requesting an increase in staffing because of grey nomads or suggested this was necessary, irrespective of their recruitment issues. These services were all small (see Table 1), with clinical and administrative staff covering a range of functions. Any presentation that tied up a member of staff impacted these services. In participants’ view, whilst grey nomads had some presence in their EDs, this was not anything out of the ordinary. Only one manager saw grey nomads specifically as having an impact, but even she described this as, “*they impact normally…” [HCP5];* that is, no different to other presentations. This was further explained:

*“Ah look, anyone that is significantly unwell impacts on a small rural site because we have one registered nurse on per shift and that’s the only person in this facility who can triage so the triage can take them away from something else they would be doing...” [HCP1].*

Resource-intensive episodes necessarily resulted in significant disruption to routine services, regardless of whether the patients were local people or grey nomads. Examples were provided where local people had experienced serious illness and major traumas such as cardiac events and farm accidents and where presentations by travellers were similarly resource-intensive. For example in one ED a grey nomad died from a ruptured abdominal aortic aneurysm; motor vehicle and caravan accidents were also recounted (see Supplementary file 1, Case Example Two). Grey nomad presentations were reported as most commonly men attending with chest pain or known cardiac disease. Medication-related attendances were almost as frequent, when travellers had left their medications at home, had run out of pills, or didn’t have a prescription to refill. Accidents and injuries also featured, such as:

“ *dropped the tow ball or the trailer on their leg [HCP8]; …sepsis, fractures, ankles, lots of falling over including the curb, that’s quite common; ankles or wrists, just those accidental things that happen…” [HCP5].*

One participant gave accounts of grey nomads presenting to the facility during winter for a dose of annual influenza vaccination (and other vaccines) and explained that this site is allocated additional flu vaccine doses for this population. The consistent picture was that participants applied an inclusive definition of community: they did not differentiate presentations by grey nomads, who were not perceived to be a particular burden, and there was no difference in the care they received from that of local people.

### Met and unmet expectations

This theme related to the expectations of some grey nomads of the services they anticipated would be available, what services are normally delivered and what can be provided where staff go out of their way to meet peoples’ needs.

The majority of presentation anecdotes illustrated travellers presenting with needs that were within scope for the facilities either to address directly or arrange care delivery elsewhere. However, services that are taken for granted in metropolitan areas are not always available in these remote sites. Some travellers or even urban service providers were not aware of this.

*“We have people from the city hospitals ring us and want to speak to our CT person or our pharmacist. So that would be me, and that would be me, and that would be me. We don’t have a CT scanner, we only have an xray machine. It’s not just travellers, it’s other people in general… they don’t know” [HCP11].*

*“…So you know most of them are great but there’s the odd few who will say “fly me out, what do you mean?” “We don’t do that here, we’ve got to fly you out”, and most of them handle it well but being several hours from a Base hospital, I think some of them don’t necessarily get the distance and what a rural hospital does…” [HCP6]*

As specialty services are not necessarily available, patients may have to be flown out to larger centres. One participant recounted a conversation with a patient who seemingly did not understand the available services or the requirement to be flown to another hospital, saying:

*“… do I have to?* (be flown out)” *[HCP3].*

Where services were available, this could be because people were multi-skilled, for example, where wardsmen and nurses were qualified to take X-Rays.

Sometimes staff rose to the challenge even where expectations were not realistic. In Case Example Three (see Supplementary file 1), despite unrealistic expectations, staff were able to arrange what was asked. In Case Example Four (see Supplementary file 1), advanced practice skills were employed by a single nurse in a Primary Healthcare Centre (PHCC), not just to manage seriously decompensated diabetes but also to understand the mental state of vulnerable people in high-risk situations. In these instances, whilst the expectations were described as unrealistic, they were met.

Grey nomads’ unrealistic expectations could extend beyond the services to encompass the countryside and their travel within it. Not all travellers had an awareness of the vastness of the country, the remoteness, the distance between towns, and the need for this to inform their level of preparedness for their travel and their health maintenance.

“*The towns are an hour apart but … some people just don’t understand the distances” [HCP 11].*

### Travel preparations

Participants described grey nomads with various levels of understanding and preparation for their travels. They were more generally characterised as educated and informed, knowing their doctors and conditions, having a care plan, discharge summary or some type of health information pack, and a list of their medications and scripts. Whilst MyHealthRecord (Australian online national voluntary health record) was viewed as potentially useful, not all managers knew how to access it and not all grey nomads had a digital record. Those with chronic illness were described as the most prepared, reflected in having plenty of medication and a medical history from their GP.

*“…The travellers that come seem to be well prepared and have GPs and things which is always good so maybe that’s why we don’t see as many as we could, they might take a little bit better care of themselves I’m not sure cause they are planning to be away so maybe they organise that stuff….*” [HCP 1]

*“…Those who are well prepared usually have chronic health issues; have plenty of supplies such as food water spare tyres; know where they are going; have plenty of medication; letter of introduction from their GP and bring a medical history with them”.* [HCP10]

However, this wasn’t always the case. There were other instances, albeit not so commonly described, where forward planning and preparation was lacking, especially for problems or sudden ill-health:

*“…Some just get in their car, pack their car up and the car might be 20 years old and off they go. That can be male or female because we do have single male travellers as well not prepared at all. They might only have a little bit of water with them, not even have an idea of the distance between towns...”* [HCP 8]

*“…Some are, some aren’t. Some haven’t got a clue and don’t understand what resources are and are not available in the outback...”* [HCP10].

In one facility the story was recounted of a male traveller and his wife who presented to the ED to have blood collected for pathology tests every couple of days. Although not considered a burden on the service, the participant expressed concern that this man was in such a remote location with a compromised immune system, and questioned how prepared he was, should he become ill enough to warrant admission to hospital. The concern was about the patient, not the impact on the service. Another manager recounted how a man and his wife towed a caravan to the hospital but had no contingency plan when the man became ill and required retrieval to another hospital: the wife could not drive let alone tow a caravan (see Supplementary file 1 Case Example Five). Another manager described a couple who presented where the husband, who was the driver towing the caravan, had dementia and his wife did not drive. In some instances, travellers’ preparedness was linked to their expectations of the support available to them in remote Australia: expectations that were not always realistic. Poor preparedness could reflect a failure to think through the implications of remote travel. In other cases, however, good preparedness seemed an extension of usual self-management: of routine accommodation of health needs within their lifestyle, whether in a house or caravan.

“…I do find that people who travel are organised, they’ll come with their list of medications and their medical conditions, all their paperwork together and hand it to you..”. [HCP6]

## Discussion

This study aimed to explore the utilisation of healthcare services in remote locations in Australia by grey nomads, from the perspective of healthcare professionals working in these settings. The interview findings illustrated the strong service and community ethos of managers. This meant they were both inclusive and flexible in how they worked and how their facilities functioned in order to meet the holistic needs of all their patients, sometimes to a much broader extent than would be usual in urban settings. Grey nomads were viewed as members of the community served, for whom care was provided. Whilst they were reported to comprise only a small proportion of remote service workloads, their needs were regarded no differently to those of local people. Very different service configuration and availability were reported in the remote areas compared to metropolitan areas. A mixed picture was presented of travellers’ expectations, with some examples where ignorance of what can be provided drove unrealistic expectations. Similarly, their levels of preparedness varied. Most but not all, and particularly those with chronic illness, were described as well-prepared for their travel and for illness self-management and health maintenance during their journeys.

This study adds new knowledge in relation to grey nomads’ use of remote health services from the perspective of service providers. These managers regarded grey nomads as part of the community served by facilities and saw no distinction between meeting the needs of the transient group and local populations. The reported frequency of grey nomad presentations varied but was not considered a significant addition to workload or a burden. Grey nomads were described as generally well prepared for travel in relation to the self-management of their health. Their expectations of what services were available were occasionally unrealistic but were usually within scope and able to be met.

Only two previous studies, from Tate, Mein [7] and De Bellis, Hill [22], have surveyed health care professionals. The first study to seek the views of healthcare professionals [7] was undertaken in 2006 in the remote Kimberley Region of Western Australia. This examined, and raised as an issue, the potential “burden” of disease and (un)preparedness of older travellers visiting remote Australia. Findings included that only half the participants had sufficient medication for their trip, only 19% had a list of long-term medications and 9% a health summary. These authors concluded that the presence of chronic illness and the absence of health documentation indicated that older travellers were poorly prepared for such travel and therefore a burden on remote health infrastructure. In the other study seven credentialled diabetes educators working in rural and remote South Australia were interviewed in 2018 about their experiences of providing care for grey nomads with diabetes [22]. By contrast, these educators described the grey nomads as mostly well-prepared although it was the small proportion with poor diabetes education and self-management skills who were more likely to need their help. They appreciated the diversity that these travellers brought to their working lives, and there was no suggestion of “burden”.

Other studies asked grey nomads about their health service usage, and reported a mixed picture. Acute events and medical emergencies were described as few [5], or affecting 5% of travellers [23]. Health scares were more common, reported by 14% [23] and by 32% of men and 21% of women in the previous 2 years, and treated locally in regional hospitals or by GPs [24]. From online survey respondents, Halcomb, Stephens [8] reported that 42% had visited a General Practitioner and 23% an ED. Of the total sample, only 7.6% had sought medical attention to obtain a prescription and 18% had got prescriptions filled while travelling. It is difficult to determine if the mixed results of these sparse and heterogenous studies constitute high usage or imposition on health services.

In the current study travellers were described as generally well prepared, with one participant noting that with the internet people have more access to relevant information, contributing to their better preparation. Studies reviewed by Yates, Perry [5] revealed that themes predominantly described grey nomads as experts in self-management and well-prepared for travel. Raven [13] noted the largely anecdotal nature of the evidence of Tate, Mein [7] for their burden on rural/ remote services, but this concern was nonetheless picked up and repeated in later studies. These concerns, derived from surveys and interviews with grey nomads, contrasted the views expressed by the managers interviewed for this study and the predominant view of the diabetes educators interviewed by De Bellis, Hill [22] where grey nomads were regarded simply as people needing healthcare and no different than anyone in their local communities.

A number of reasons may explain, at least in part, this discrepancy in findings. Tate, Mein [7] interviewed primary care doctors and nurses, whereas hospital managers were interviewed for this study. The context for the two studies was different, for example in terms of staffing models, workload, funding and the care provided. Changes in the age and health literacy characteristics of cohorts of travellers during the sixteen years reflect changes in the general population, which is both ageing and better informed. The studies from Halcomb, Stephens [23], Calma, Halcomb [9] and Stevens [11] targeted grey nomads with chronic illness via an online survey. Altogether, the studies that comprise this body of knowledge vary widely in terms of time, setting and participants and this diversity likely contributed to the varied findings. The current study makes a significant contribution based on the different context of the perspectives of the hospital managers, and the depth of information they provided.

Whilst this study is limited in deriving from a small sample of hospital managers, data saturation was achieved for these interviews. Further, participants worked for the public health providers of two of Australia’s three most populous states, on a popular remote travel route. It is regrettable that no hospital activity data were available to support participants’ reported experiences.

## Conclusion

In conclusion, grey nomads were not perceived to burden remote health services. Managers applied an inclusive definition of community, regarded travellers as part of this community when accessing health services, and their contribution to the local towns was welcomed. Traveller numbers have been increasing over recent decades and the COVID-19 pandemic has resulted in significantly more people buying caravans, campervans and other mobile accommodation and travelling domestically in Australia. Particularly in light of these increasing numbers, further research is required to examine the rates and details of presentations by domestic travellers including grey nomads to remote health services, and to determine what proportion may be sustained rather than transient. Routine data detailing traveller presentations should be collected to support appropriate ongoing resourcing of healthcare facilities in remote areas and to better inform service policy and planning.

Despite the different perspectives described by the healthcare professionals in papers written up to sixteen years apart, the recommendations of earlier studies remain relevant. These include travellers having a health summary including medical history, current problems, medications and allergies; this could now be achieved with a more comprehensive uptake by the population and utilisation in health services of MyHealthRecord.

The healthcare services that can be provided in remote areas needs to be better understood by urban travellers and their regular healthcare providers. This information could be more widely publicised in order to support consistent planning and preparation for travel. It was widely acknowledged that people with chronic illness were better prepared for travelling. Perhaps people who consider themselves healthy and do not have a chronic illness may not anticipate and prepare for acute illness that requires healthcare support. Further research is clearly required to understand the diversity of travellers’ needs. Overall, however, the dominant picture was one of healthcare services and managers operating with flexibility and inclusivity, committed to serving their communities, including grey nomads.

## Supplementary Information

**Additional file 1.** 

## Data Availability

The datasets generated and analysed during the current study are not publicly available as they are part of an ongoing doctoral study but are available from the corresponding author on reasonable request.
