# Supplementary material for: ‘Grey nomad’ travellers’ use of remote health services in Australia: a qualitative enquiry of hospital managers’ perspectives
Source: BMC Health Serv Res. 2022 Feb 5;22:151. doi: 10.1186/s12913-022-07580-8 (PMC8817147; doi:10.1186/s12913-022-07580-8)
Supplement: Supplementary file 1 — Additional file 1. [file 12913_2022_7580_MOESM1_ESM.docx]

**Supplementary File One**

**Case Example One: Caring for their community**

**HCP4**: “…There was a man that was travelling on his own; we ended up looking after his dog for a week because he was actually quite unwell, he got sent off to Sydney so his caravan was kept here. And he had a little dog. He’d lost his wife in the last six months before he arrived here so he left his caravan here and we adopted the dog.

**Researcher**: Who looked after the dog?

**HCP4**: One of the nurses

**Researcher**: How long did she have the dog for?

**HCP4**: A week; the whole time he was away

**Researcher**: Where did you keep the caravan?

**HCP4**: Out the back of the hospital and, he was really worried because he had a port-a-loo and he said that it needed emptying, the maintenance man fixed all that up for him….”

**Case Example Two: Travellers’ impact on services**

**HCP2: “…**We have had presentations here and lots of the presentations are around cardiac stuff, so we’ve had to fly them out. When we’ve had them it’s always difficult because their poor wife is here, they’re stuck lots of times, we’ve stored the caravans out the back under the cameras and we’ve allowed the partner to stay here in the hospital with them or they’ve slept in the car park around the back. Look yeah it’s not an issue. It’s the same as anybody sometimes you just work that bit harder when it is a grey nomad or anybody that comes here that doesn’t come from here you take all those things into consideration maybe they’ve got an animal or they are going to be stuck out at, I know that one of the ones we had last year when the season was happening they were with a group of people and they got to town and they were actually on the start of their journey they got here and he had some cardiac issues so we had to fly him out. It was really traumatic for her because she couldn’t drive. And so the others were leaving the next day and we just sort of helped, we got her on the flight which was good which is sometimes an advantage if we can get them on the Air Ambulance flight….”

**Case Example Three: Unrealistic Expectations**

**HCP11**: “…We do have quite a few come through and use our resources quite readily; not just women, men as well. The grey nomads in particular, and the expectation of what services can be delivered out this way is far higher than what is actually able to be serviced.

**Researcher**: What do they expect?

**HCP11**: I’ll give you an example. We had a person come through here … who was on a trial study for cardiac myopathy. She was from Victoria and she needed to take it on a daily basis. She went to the pharmacist in town first and asked them to get it, the drug, and the pharmacist couldn’t get it because it is off the approved medication list so the pharmacist directed her to us because we provide a small hospital pharmacy through our regional hub so we rang the largest facility pharmacy and the pharmacist just said that was above her pay grade as well so we went to the State-wide chief pharmacist and he said he’d look into it. Some trial drugs they can provide, others they can’t, this one they couldn’t. The actual pharmacy annual budget was around $16,000 at the time and the drug was $24,000; it was worth more so we said we just couldn’t do it and the lady went off, “*My specialist said I can get this drug anywhere in Australia…*” and we explained that you probably can but you would have to pay and she said that’s not what she had been told. We called her specialist and the specialist had no idea they were going to travel and there was no plan of care for what the person required observationally or medications. Our town is small isolated, the water, because of the high mineral content, reacts with some drugs including another one she was prescribed. This person had enough drugs to get her to the larger town further on that had a franchised pharmacy of the chain where she gets her trial drug dispensed. There were only two places in the whole of Queensland that had these franchises at that time that could access the drug through the trial. It became a logistical nightmare for us so now we set out on our hospital website, the five primary health centre websites the shire council’s websites, that if you are a traveler coming into our shire make sure you have enough medication to last you.

**Researcher**: How many hours would you have been tied up you and your staff as well?

**HCP11**: What tied me up was the phone calls, finding the right person to talk to was probably the hardest but also the pharmacist, the expectations of those people we contacted with what we can provide including her specialist. He did not understand why she couldn’t get the drug from us…”

**Case Example Four: Expectations Met**

**Researcher**: Where is your experience of grey nomads?

**HCP12**: “…mostly rural and remote out the “Back O’Bourke” I always call it and um where there’s a single nurse post in Primary Healthcare Centres. They see the blue hospital sign and they’ll come there because we often have Royal Flying Doctor Service [RFDS] flying in. For example, we had a couple of sick grey nomads come through and we have the RFDS fly in once a fortnight to do a day clinic but the rest of the time the nurse is dealing with whatever the presentations are. One grey nomad was in diabetic ketoacidosis, so somebody who had vomiting and diarrhoea, and they had a basal insulin pump on that wasn’t working appropriately because they were sick and the pump was trying to measure what their glucose levels were and supply the appropriate insulin but realistically we had to remove the pump and we give them some education around needing the pump, measuring your own sugar levels and we had to put an IV in and give them some fluids….

[Another] one was a diabetic man as well and he had, first of all his wife found him unconscious in the caravan and when we went there, he was OK but then after that we took him to the hospital and he was flown out. But then he came back and he had an infection. He was a really bad diabetic. But he understood that and he said “yeah yeah I know all that but I’m going to travel before I die and I’m just going to do that”… And I guess because I have been working independently for such a long time I have a different attitude to a hospital because I have found with my nursing staff they have a totally different attitude about grey nomads “Oh that grey nomad he’s forgotten what tablet he takes, they don’t understand their diabetic regime, you know’’, so I think it’s hospital based perception of what a grey nomad looks like and you heard that opinion but in my opinion I find that they are a lot more educated than they used to be and I’ve seen them way back when, when they didn’t even have a medication list, didn’t even know what their medications were. Now they have a pretty good understanding of their illness and they can actually tell you about it. In a lot of cases clients have a better understanding about their health then we do because they have lived with it and our best defence is to listen to what they’ve got to say. And we are an aging population so we are going to get more of them you know so it’s inevitable that we will be faced with the challenges of aging’’.

**Case Example Five: Traveler Preparation**

And then it is challenging for the ladies left behind, when we fly out their partner and now they are left with, “I have a caravan, I don’t tow, I don’t drive”.

**Researcher:** You are still finding women saying that?

**HCP9:** Absolutely, so no preparation or forward thinking about what could happen or what would happen and how they would manage

**Researcher:** So do you take that on?

**HCP9:** We get in contact with Queensland Police Service. If we have to**,** we obviously help them make all the phone calls they do. So if they left their caravan somewhere we say, if you let the police know they’ll make sure they keep an eye on things. Because we don’t have after hours social work support. They visit once a week from [larger town] as an outreach. We take on the social work support as much as we can so we can support them.
